# Primary non-refluxing megaureter: Natural history, follow-up and treatment

**DOI:** 10.1007/s00431-024-05494-7

**Published:** 2024-03-05

**Authors:** Giulio Rivetti, Pierluigi Marzuillo, Stefano Guarino, Anna Di Sessa, Angela La Manna, Anthony A. Caldamone, Alfonso Papparella, Carmine Noviello

**Affiliations:** 1 Department of Woman, Child and of General and Specialized Surgery, Università degli Studi della Campania “Luigi Vanvitelli”, Via Luigi De Crecchio 2, 80138 Naples, Italy; 2 Division of Urology, Warren Alpert School of Medicine at Brown University/Hasbro Children’s Hospital, Providence, RI USA

**Keywords:** Primary non-refluxing megaureter, Urinary tract infections, Continuous antibiotic prophylaxis, Ureteral replantation, High-pressure balloon dilation

## Abstract

Primary non-refluxing megaureter (PMU) is a congenital dilation of the ureter which is not related to vesicoureteral reflux, duplicated collecting systems, ureterocele, ectopic ureter, or posterior urethral valves and accounts for 5 to 10% of all prenatal hydronephrosis (HN) cases. The etiology is a dysfunction or stenosis of the distal ureter. Most often PMU remains asymptomatic with spontaneous resolution allowing for non-operative management. Nevertheless, in selective cases such as the development of febrile urinary tract infections, worsening of the ureteral dilatation, or reduction in relative renal function, surgery should be considered.

*Conclusion*: Ureteral replantation with excision of the dysfunctional ureteral segment and often ureteral tapering is the gold-standard procedure for PMU, although endoscopic treatment has been shown to have a fair success rate in many studies. In this review, we discuss the natural history, follow-up, and treatment of PMU.

**Table:** 

**What is Known:** *• PMU is the result of an atonic or stenotic segment of the distal ureter, resulting in congenital dilation of the ureter, and is frequently diagnosed on routine antenatal ultrasound.*	
**What is New:** *• Most often, PMU remains asymptomatic and clinically stable, allowing for non-operative management.* *• Nevertheless, since symptoms can appear even after years of observation, long-term ultrasound follow-up is recommended, even up to young adulthood, if hydroureteronephrosis persists.* *• Ureteral replantation is the gold standard in case surgery is needed. In selected cases, however, HPBD could be a reasonable alternative.*

**What is Known:**

*• PMU is the result of an atonic or stenotic segment of the distal ureter, resulting in congenital dilation of the ureter, and is frequently diagnosed on routine antenatal ultrasound.*

**What is New:**

*• Most often, PMU remains asymptomatic and clinically stable, allowing for non-operative management.*

*• Nevertheless, since symptoms can appear even after years of observation, long-term ultrasound follow-up is recommended, even up to young adulthood, if hydroureteronephrosis persists.*

*• Ureteral replantation is the gold standard in case surgery is needed. In selected cases, however, HPBD could be a reasonable alternative.*

## Introduction and definition

The term megaureter is used to describe a dilation of the ureter ≥ 7 mm [1]. This definition does not correspond to a precise diagnosis nor involves any etiological evaluation as the ureteral dilation is common in many uropathies such as vesicoureteral reflux (VUR), duplicated collecting systems, ureterocele, ectopic ureter, or bladder outlet obstruction as in posterior urethral valves. Megaureter can be divided in primary and secondary, depending on the nature of the dilation which can be intrinsic or related to another urinary tract pathology [2]. Megaureter is also classified as obstructed, refluxing, obstructed and refluxing, or neither obstructing nor refluxing, using the international classification of Smith [3].

When the dilatation is not related to secondary causes, it is termed primary non-refluxing megaureter (PMU). PMU is frequently diagnosed on routine antenatal ultrasound (US) accounting for 5 to 10% of all prenatal hydronephrosis (HN) cases [4, 5]. PMU grade classification is not codified, but a distinction in mild (7–10 mm), medium (10–15 mm), and severe (>15 mm) may be clinically useful. PMU is more common in males and most often involves the left ureter, although 25% of cases are bilateral. When unilateral, 10–15% of cases have an absent or dysplastic contralateral kidney [6]. In this review, we discuss the natural history, follow-up, and treatment of the PMU in order to give to general pediatricians a comprehensive and practical overview.

## Strategy and selection criteria

We searched PubMed and relevant specialty journals, from 1983 to 2023. We used the search terms “primary non-refluxing megaureter” or “megaureter” and “urinary tract infections” or “continuous antibiotic prophylaxis” or “ureteral replantation” or “high-pressure balloon dilation.” We choose principally publications from the past 15 years without excluding commonly referenced, relevant, and influential older publications. We searched only articles in English or those translated into English. We also searched the reference list of articles identified by this strategy and selected those we judged relevant for this paper on the basis of the abstract review. We included observational studies, retrospective studies, meta-analyses, and case reports. Book chapters, guidelines, review articles, and editorials were also included in the search strategy to provide a more complete and wide vision of the topic.

## Baseline evaluations

US is the basic investigation in the diagnostic path of PMU and is useful to monitor ureteral and renal dilation over the time [7].

In addition to the information on ureteral diameter, US can be useful to obtain information about possible abnormalities of kidney parenchyma (i.e., echogenity, cystic changes, parenchymal thickness), anterior-posterior renal pelvis diameter, possible dilation of peripheral calyxes, and bladder abnormalities [8]. The best measurement site of the ureter in children with megaureter using US is the distal ureter above the vesico-ureteric junction [7]. This is the area where the ureter is most dilated and shows active peristaltic waves [9].

The distal ureters are best evaluated in the supine position. Non-dilated ureters are visualized on occasion; however, dilated ureters should not be missed and should be examined in transverse and longitudinal planes [10]. If the ureter is dilated, the transverse diameter should be measured and documented, along with the degree of dilatation seen [10]. This offers the clinician an overall impression of the degree of dilatation and is valuable for comparison in serial scans [10].

While it is true that PMU often resolves spontaneously [11], considering that PMU is also related to vesicoureteral reflux or posterior urethral valves, it is important to accurately define the PMU diagnosis ruling out other conditions associated with the presence of megaureter. This, in fact, could orientate clinical approach with a wait-and-see approach in case of VUR [12] or cystourethroscopy in case of posterior urethral valves suspicion on voiding cystourethrography (VCUG) [13].

Of paramount importance is the accurate interpretation of urethral cystography, as evaluating even the indirect signs enhances the diagnostic performance of cystography compared to valves [14].

In patients who have been diagnosed with PMU antenatally, a postnatal US scan should be performed. An initial normal postnatal US may be misleading [8] due to the low intravascular volume in the neonate resulting in low urine production. In fact, 21–28% of children with prenatal urinary tract dilation have a normal initial postnatal US and in 45% of these patients, an abnormal US at follow-up may be seen [15]. Moreover, 5% of the patients requiring surgery for obstructive uropathies had a normal US at 1 week of age but an abnormal US at 1 month of age [16]. In case of prenatally detected PMU, an early postnatal US after 48 h of age should be performed to select cases to consider specific and early treatment [8]. If the initial ultrasound is normal, a repeat US at 1 month of life is recommended. According to the British Association of Paediatric Urologist guidelines in patients with megaureter, a VCUG should be performed to exclude the presence of vesicoureteral reflux or bladder outlet obstruction such as posterior urethral valves, especially in patients with bilateral or severe unilateral megaureter [17].

Moreover, all the children presenting with PMU ≥ 10 mm should undergo Tc99mMag3 scintigraphy at 4–6 weeks of life to assess relative renal function.

Continuous antibiotic prophylaxis (CAP) could be suggested in all the newborns with megaureter until a definitive diagnosis is made. In case of diagnosis of PMU, CAP in the first 6–12 months of life could be recommended especially in patients with ureteral diameter > 10 mm [11, 17], as patients with ureteral diameter > 10 mm not receiving CAP have significantly higher rates of febrile urinary tract infections (fUTI) compared to those on CAP (53% vs 21%) [11].

In a recent study, hyponatremia, observed in 2.8% of patients during the first episode of fUTI, was found to be associated with mild pelviectasis in imaging; however, it does not imply an elevated need for additional tests to evaluate urinary tract malformations [18].

The main risk factors for the development of fUTI are the presence of phimosis and the absence of CAP, while ureteral tortuosity and dilatation have not been demonstrated to be independent risk factors for fUTI [11].

Nevertheless, over a 3-year period, the proportion of children with resistant UTI on CAP doubled, with those having congenital anomalies of the kidney and urinary tract (CAKUT) being more susceptible to resistant infections, highlighting the necessity for investigating and advancing alternative non-antimicrobial prophylaxis options [19]. Supporting this hypothesis in another CAKUT, the PREDICT study showed that in infants with grade III, IV, or V VUR and no history of UTIs, CAP demonstrated a modest yet significant advantage in averting initial UTIs, despite an elevated presence of non-*Escherichia coli* organisms and antibiotic resistance [12].

## Outcome and natural history

Prior to the routine use of prenatal US, most children with PMU were diagnosed only after the development of symptoms such as fUTI, hematuria, and abdominal pain [1]. On occasion a newborn may present with a palpable abdominal mass (kidney or ureter) [20, 21]. The widespread use of prenatal US allowed for prenatal identification of PMU. Many of these cases have been observed to remain asymptomatic with spontaneous resolution allowing for non-operative management [11, 22–26].

Resolution is most often early, within the first 2 years of life [1, 11], however, it has been reported up to 5 years and in some cases into young adulthood [11]. However, US is a very variable technique with different interpretations based on the operator and on the patient’s fluid intake. For this reason, it could be reasonable to evaluate the ureter diameter with physiological bladder filling (expected bladder capacity for age could be calculated with the following formula: [age (yrs) + 1] × 30 mL [27]).

In the current literature the spontaneous resolution rate generally ranges between 34 and 88% [28–30].

Of all patients with PMU, approximately 24% will require surgical intervention, particularly those with a mean ureteral diameter of 17 mm or greater, while the remaining 76% will resolve spontaneously in a median of 19 months [11]. An independent variable which is considered as a predictor of spontaneous resolution is ureteral dilation <11 mm at baseline, as these patients have been shown to be more likely to resolve within 24 months of age, while those with ureteral dilation ≥ 14 mm are instead more likely require surgical intervention [1]. Moreover, a nonobstructive washout pattern and prenatal or neonatal presentation are predictors of spontaneous resolution [31].

Since potential long-term complications are described in the current literature [32], long-term US follow-up is recommended at least until puberty, depending on the postoperative ultrasound appearance, as symptoms can develop even after years of observation [32].

## Follow-up

The purpose of follow-up is (i) to verify the resolution of PMU; (ii) to identify complications and worsening (evaluating indications to surgery); and (iii) to reduce painful procedures, exposure to radiation, and economic costs by accurate risk stratification.

**Fig. 1:**
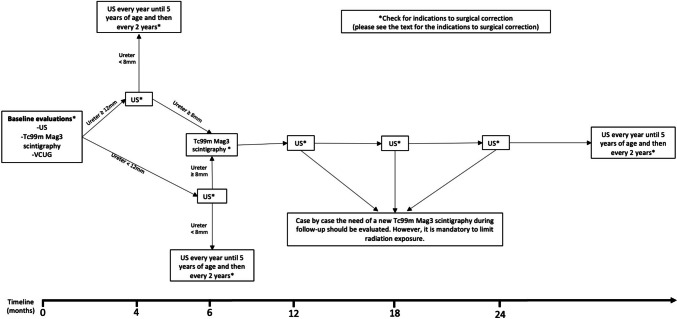
Follow-up flowchart for PMU. US, ultrasound; VCUG, voiding cystourethrogram; Tc99m Mag3, mercaptuacetyltriglycine

In order to give a practical guidance to the readers, we present the protocol adopted in our center (Fig. 1). After the baseline evaluations to confirm the PMU diagnosis, if there is no need of early surgical intervention (please see “Indications for surgery” section) and the spit renal function is > 40%, the patient could undergo follow-up US at 3 and 6 months. A repeat Tc99mMag3 scintigraphy 6 months later is indicated if there is no improvement or if there is an increase in the hydronephrosis, in the absence of symptoms.

Repeat Tc99mMag3 scintigraphy could be considered on a case by case basis, as the dilation is followed. However, if surgical indications are clearly present, such as recurrent febrile UTIs, significantly increased dilation, or the development of symptoms, a radiation sparing approach without renal scintigraphy should be considered. The exposure to excessive radiation, especially if early in life, has been associated with an increased risk of cancer during the life of patients [33].

## Definition of PMU resolution

PMU resolution is defined by having stable 10 mm or less anteroposterior diameter of the renal pelvis and/or SFU hydronephrosis grade 2 or less, less than 8 mm ureteral dilatation [11]. Nevertheless, this condition needs some attention during the follow-up with US every 1–2 years (Fig. 1).

## Indications for surgery

The main indications for repair of PMU include fUTI, kidney stones, abdominal symptoms, megaureter diameter > 10 mm with split renal function < 40% on initial Tc99mMag3 scintigraphy or a split renal function with delta > 10% on subsequent Tc99mMag3 scintigraphy, or worsening of dilatation on repeat ultrasounds (> 14 mm) [11, 34]. However, we would specify that a reduced split renal function may be also related to a kidney dysplasia associated to PMU [35–37]. To discern between these conditions, it could be useful to interpret together kidney ultrasound and findings of Tc99mMag3 scintigraphy. If the split renal function is reduced and on US the cortico-medullary differentiation is abnormal and/or the kidney is small, it is more probable a congenital dysplasia. On the other hand, if the kidney echotexture is normal and the split renal function is reduced or if the split renal function decreases in the next follow-up, an obstructive acute damage is more likely.

While a strict association between megaureter diameter > 14 mm and surgery exists [1], a cut-off to suggest surgery on the basis of the increase of dilation at US has not been established. However, it is recommended, however, that an increase in ureteral dilation is confirmed by repeat US before recommending surgery, as hydronephrosis is influenced by the degree of bladder fullness and the state of hydration.

Recurrent fUTIs and ureter > 14 mm are the only variables independently associated with surgery [1], with fUTI the most frequent indication for surgery [11]. However, in the 85% of patients undergoing surgery there were at least two indications [11].

Some authors include an “obstructive” wash-out on Tc99mMag3 scintigraphy (characterized by an accumulation of the isotope within the kidney and a drainage curve that continues to rise even after change of posture or diuretic to encourage drainage [6]) as an indication for surgery [31]. However, this criterion is inconsistent and not sufficient alone to proceed with surgical intervention, as the measurement of washout across a dilated ureter into the bladder is technically problematic.

While there is consensus regarding surgical indications [11], the final decision to proceed with intervention relies on an open discussion with the family regarding goals and complications.

## Surgical approaches

The laparoscopic approach for PMU in children involves using minimally invasive techniques to correct the condition [38]. This typically includes creating small incisions in the abdomen and using a laparoscope to visualize and manipulate the affected area [38]. Laparoscopic techniques offer advantages such as shorter recovery times and reduced scarring compared to traditional open surgery [38]. Robotic surgery, akin to laparoscopy, is also a widely utilized technique which involves a robotic system controlled by the surgeon to deliver enhanced precision and a broader range of motion for the instruments, offering advantages in complex procedures and enabling more intricate maneuvers [39].

Ureteral reimplantation with excision of the distal ureteral segment, with or without ureter remodeling, is considered the gold-standard procedure for patients with PMU, with a well-documented success rate (90 to 96%) [40].

However, in younger patients (< 12 months of age), the disparity between a very dilated ureter and a small bladder can make the procedure problematic and could result in complications (in about 4–25% of cases), such as secondary obstruction, secondary VUR, or transient bladder dysfunction [17]. For these reasons, when a surgical treatment is needed in the first months of life, temporary diversion, such as cutaneous ureterostomy, refluxing reimplantation, or double-J stenting, can be used [40].

In this regard, Sang et al. described the refluxing ureteral reimplantation as a viable method for temporary urinary diversion in neonates and infants experiencing severe hydroureteronephrosis is particularly in cases with uncontrolled urinary tract infection and/or compromised renal function [41]. Moreover, this procedure ensures efficient renal drainage, permits a single-stage reconstruction 12 to 18 months post-initial operation, and mitigates inconvenience for the child and their family [41].

Cutaneous ureterostomy remains the preferred procedure for severe acute septic complications of PMU, especially in the very young patients [42].

Endoscopic treatment involves dilatation of the atonic or stenotic distal ureteral ring with a high-pressure balloon dilation (HPBD). It was initially proposed in the patients < 12 months of age as a transitional improvement procedure until reimplantation [43] could be safely undertaken, and later shown to be successful in some cases avoiding open surgery [44].

The short- and long-term results with the endoscopic approach were confirmed by many groups concluding that the procedure was achievable with success rates between 67 and 95% [45]. If endoscopic treatment fails, open reimplantation can still be done. A complication rate of 23 to 60% following surgery was documented, primarily consisting of temporary hematuria, urinary tract infections, and issues related to stent movement or intolerance [46].

The short- and long-term results with the endoscopic approach were confirmed by many groups concluding that the procedure was achievable with success rates between 67 and 95% [45]. If endoscopic treatment fails, open reimplantation can still be done. A complication rate of 23 to 60% following surgery was documented, primarily consisting of temporary hematuria, urinary tract infections, and issues related to stent movement or intolerance [46].

The pros and cons of HPBD are indicated in Table 1 [42, 45, 47, 48]. However, the final decision about the surgical approach should be evaluated by the surgeon case by case. After HPBD patients should undergo precise clinical follow-up: a clinical evaluation and an US scan should be performed at 3, 6, and 12 months after the removal of the stent, and then every 6 months. If after 2 years the patient is still asymptomatic and the diameter of the distal ureter is < 7 mm, the follow-up can be stopped and an US scan could be performed every 3–5 years. Moreover, a Tc99mMag3 scintigraphy could be considered after 6 months from the removal of the stent, especially if there is no change in the degree of hydronephrosis or ureteral dilation.

**Table 1:** Pros and cons of HPBD

**Pros**	**Cons**
Avoids the development of bladder injury, the manipulation of the distal ureteral vascularization and a post-operative bladder catheterization besides reducing the duration of hospitalization [29]	Use of radiation (fluoroscopy) during the procedure
Available at any age [25]	Placement of JJ stent to be removed under anesthesia after 1–2 months
	Percentage of postoperative complications (poor tolerance to JJ stent, JJ stent migration, postoperative UTI, persistent hematuria) estimated between 40 and 70% [25]
	Secondary reflux (5–27%) [28], but not high in grade, often asymptomatic and transient. This secondary reflux can be endoscopically treated, if necessary. For these reasons, post-HPBD VUR is considered clinically irrelevant and transient and post-procedural VCUG is not necessary
	Recurrence of stenosis (with the possibility to repeat the procedure using the "cutting balloon") [30]

*HPBD* high-pressure balloon dilation, *VCUG* voiding cystourethrogram, *UTI* urinary tract infection, *VUR* vesicoureteral reflux

Recently, Ripatti et al. suggested that HPBD seems safe and could serve as the primary treatment for symptomatic PMU, yet additional comparative studies are required to evaluate its efficacy in infants and its long-term effects, with the challenge remaining in accurately identifying patients who would benefit from HPBD due to the complex nature of PMU [49].

Finally, despite the extensive case studies described, the scientific evidence of endoscopic treatment of PMU is poor. Long-term follow-up data are still few and it is difficult, therefore, to determine whether endoscopic dilation is a definitive treatment for PMU. Comparative studies are needed between ureteral and endoscopy replantation to assess the role of this latter as a first-choice treatment.

## Conclusions

PMU is the result of an atonic or stenotic segment of the distal ureter resulting in congenital dilation of the ureter and is frequently diagnosed on routine antenatal US. The ureteral dilation, washout pattern, and prenatal or neonatal presentation are the main predictors of spontaneous resolution of PMU. Most often PMU remains asymptomatic and clinically stable allowing for non-operative management. Nevertheless, since symptoms can appear even after years of observation, long-term US follow-up is recommended even up to young adulthood, if hydroureteronephrosis persists. The existence of undiscovered areas in this field emphasizes the potential for future research insights (Table 2). Finally, ureteral reimplantation with excision of the pathologic distal ureteral segment, with or without ureter remodeling, is the gold-standard procedure for patient with PMU requiring surgical intervention. In selective cases, however, the HPBD could be considered as a reasonable alternative with reasonable success rates reported.

**Table 2:** Unanswered questions and future research directions on PMU

**Unanswered questions**	**Future research directions**
*Are there early predictive biomarkers of renal damage in patients with PMU?* At the present time we do not have biomarkers able to predict the appearance or the evolution of the kidney damage due to obstruction in patients with PMU.	Additional research on early predictors of kidney damage may be essential to identify patients at a higher risk of long-term consequences, warranting an early surgical approach.
*Does CAP change the outcome of PMU?* Antibiotic prophylaxis might be a sensible regimen to prevent UTI in populations who are potentially at increased risk. However, studies examining the efficacy of prophylactic antibiotics are sparse in the setting of PMU.	A randomized study about the CAP in children with PMU since birth could be useful to definitively clarify the impact of CAP on UTIs and on kidney scars. Moreover also the CAP duration should be clarified.
*Can HPBD be used as the first line treatment for persistent or progressive PMU?* If the HPBD could be the first line surgical approach for persistent or progressive PMU needs to be clarified	Since HPBD has been proven to have a reasonable success rate available at any age [25], further evidence on whether this technique could replace ureteral reimplantation, in order to reduce the rate of surgery, should be investigated.

*PMU* primary non-refluxing megaureter, *CAP* continuous antibiotic prophylaxis, *VUR* vesicoureteral reflux, *UTI* urinary tract infection, *HPBD* high pressure balloon dilation

## Data Availability

N/A.

## Abbreviations

VUR: Vesicoureteral reflux
PMU: Primary non-refluxing megaureter
US: Ultrasound
HN: Hydronephrosis
VCUG: Voiding cystourethrography
CAP: Continuous antibiotic prophylaxis
fUTI: Febrile urinary tract infections
CAKUT: Congenital anomalies of the kidney and urinary tract
HPBD: High-pressure balloon dilation

## Code availability

N/A.
